# Trends in the burden of chronic kidney disease related to high red meat intake from 1990 to 2021

**DOI:** 10.1186/s12889-025-22560-3

**Published:** 2025-04-08

**Authors:** Miaofei Dai, Haixu Guo

**Affiliations:** 1 Nephrology Department, Maoming People’s Hospital, Maoming, Guangdong China; 2 Intensive Care Unit Section 3, Maoming People’s Hospital, Maoming, Guangdong China

**Keywords:** Chronic kidney disease, Red meat consumption, Disease burden, Global health trends

## Abstract

**Objective:**

To systematically examine global trends and spatial distribution of the burden of chronic kidney disease associated with high red meat intake from 1990 to 2021 using data from the Global Burden of Disease 2021 study, providing scientific evidence for targeted prevention strategies.

**Methods:**

We assessed disease burden using age-standardized death rate and age-standardized disability-adjusted life year rate. Disease burden was analyzed by sex, age, region, and Socio-demographic Index level, while estimated annual percentage change was calculated to evaluate temporal trends.For the analysis of future trends in chronic kidney disease burden, we utilized both the Age-Period-Cohort model and the Bayesian Age-Period-Cohort model.

**Results:**

From 1990 to 2021, age-standardized death rate and disability-adjusted life year rates of chronic kidney disease linked to high red meat intake increased, with EAPCs of 1.33% and 1.07%, respectively. Males consistently exhibited a higher disease burden than females, with mortality peaking in the 85–89 age group. The age-standardized disability-adjusted life year rate in high-SDI groups (6.59 per 100,000) was significantly higher than in low-SDI groups (2.38 per 100,000). A temporary decline occurred between 2016 and 2018, and disease burden decreased among individuals over 95 years old between 2018 and 2021.

**Conclusion:**

The chronic kidney disease burden associated with high red meat intake demonstrated significant demographic and regional disparities, characterized by an overall increasing trend with some period-specific exceptions. These findings suggest the need for targeted public health interventions, particularly dietary guidance for high-risk populations such as males and residents of high-SDI groups. Future research should focus on elucidating the underlying biological mechanisms and social determinants that drive these disparities.

**Supplementary Information:**

The online version contains supplementary material available at 10.1186/s12889-025-22560-3.

## Introduction

Chronic kidney disease (CKD) represents a growing global health challenge, with its prevalence continuing to rise and contributing substantially to mortality from non-communicable diseases [1, 2]. The United Nations’ Sustainable Development Goals specifically emphasize reducing premature mortality from these diseases by 2030, underscoring the urgent need for effective CKD prevention and intervention strategies worldwide [3, 4]. Traditional risk factors for CKD have been well-documented, with hypertension and diabetes historically considered the primary contributors to disease development and progression [5]. However, emerging evidence suggests that dietary factors may play a more significant role than previously recognized. Among these dietary influences, red meat consumption has attracted particular attention for its potential impact on kidney function [6].

Multiple epidemiological studies consistently demonstrate a strong association between high red meat intake and adverse kidney outcomes, including increased CKD incidence and accelerated disease progression [7]. The underlying mechanisms linking red meat consumption to kidney damage are multifaceted and complex. Excessive red meat intake can alter gut microbiota composition and increase intestinal permeability, facilitating the translocation of uremic toxins into the bloodstream. These toxins subsequently exacerbate oxidative stress and inflammatory processes, accelerating kidney function decline through various pathophysiological pathways [8].Additionally, diets rich in red meat have been linked to metabolic disturbances, including increased production of advanced glycation end products and pro-inflammatory cytokines, which further contribute to CKD progression.

Despite the growing body of evidence connecting red meat consumption to CKD, a critical knowledge gap remains in quantifying its global burden across different populations and regions. This limitation impedes the development of targeted public health strategies and evidence-based dietary guidelines specific to diverse demographic and geographic contexts. Furthermore, the absence of robust predictive models hinders our ability to anticipate and mitigate the potential health consequences of evolving dietary patterns worldwide. As global eating habits shift, a clearer understanding of the relationship between red meat consumption and CKD burden becomes essential for effective public health planning and intervention.

This study aims to address this knowledge gap by systematically analyzing the global spatiotemporal trends in CKD burden attributable to high red meat intake from 1990 to 2021, using comprehensive data from the Global Burden of Disease (GBD) 2021 study. The findings will provide scientific evidence to support the development of targeted prevention strategies tailored to diverse populations and regions, ultimately contributing to global efforts to reduce the burden of CKD.

## Materials and methods

### Data sources

The data for this study originates from the 2021 Global Burden of Disease (GBD) study, a comprehensive global research program coordinated by the Institute for Health Metrics and Evaluation (IHME) that quantifies health loss across 204 countries and territories. The GBD 2021 database includes estimates for 371 diseases and injuries and 88 risk factors from 1990 to 2021, integrating data from multiple sources such as vital registration systems, sample registration systems, household surveys, disease registries, and population-based studies [9–11]. (https://ghdx.healthdata.org/gbd-2021/sources).

According to the parent GBD risk factor study, kidney dysfunction is classified into four categories based on urinary albumin-to-creatinine ratio (ACR) and estimated glomerular filtration rate (eGFR). Albuminuria with preserved eGFR (ACR > 30 mg/g & eGFR ≥ 60 ml/min/1.73 m²) corresponds to CKD stages 1 and 2 in the KDIGO classification. CKD stage 3 is defined by an eGFR of 30–59 ml/min/1.73 m², stage 4 by 15–29 ml/min/1.73 m², and stage 5 by an eGFR below 15 ml/min/1.73 m² without renal replacement therapy.

In GBD 2021, a high red meat diet was defined as consuming more than the average of 0 g (95% UI 0–200) of unprocessed red meat per day. This category includes meats like pork, beef, mutton, and goat but excludes processed meats, poultry, fish, and eggs. While the study systematically reviewed the risks of high red meat consumption and refined the dose–response curve for related health outcomes, existing literature commonly defines high intake as exceeding 50 g per day [12].

### Statistical analysis

#### Descriptive analysis

A descriptive analysis of the GBD 2021 data was conducted to assess the global and subgroup-specific burden of CKD associated with high red meat intake in 2021. Metrics included incidence, prevalence, mortality, and disability-adjusted life years (DALYs), reported with 95% uncertainty intervals (UIs) based on the 2.5th and 97.5th percentiles of 1,000 draws. Subgroups were categorized by sex, age, Socio-demographic Index (SDI) quintiles (low, low-middle, middle, high-middle, and high), GBD regions, and countries.

Study design and statistical analysis.

To illustrate the geographical disparities in CKD burden linked to high consumption of red and processed meat, maps were created based on mortality, age-standardized deaths rate (ASDR), and disability-adjusted life year (DALY) rate for 2021. The association between CKD burden and the SDI was examined by region and year. To assess temporal trends, the Estimated Annual Percentage Change (EAPC) was utilized, a widely recognized metric for evaluating changes in age-standardized rates over a defined period. The calculation followed the regression model y = α + βx + ε, where y denotes the natural logarithm of the rate, and x represents the calendar year [13]. The EAPC value was derived as 100 × (exp (β) − 1), with a 95% confidence interval (CI) determined using the regression model. A positive EAPC alongside a lower 95% CI bound above zero indicated an increasing trend, whereas a negative EAPC with an upper 95% CI bound below zero signified a declining trend [14]. Future trends in CKD burden from 2022 to 2046 were projected using two models. The Age-Period-Cohort (APC) model estimates incidence rates by incorporating age, period, and cohort effects through the equation: log(λijk) = µ + αi + βj + γk, where λijk represents the expected incidence rate, µ is the intercept, and αi, βj, and γk capture the effects of age, period, and cohort, respectively [15]. The Bayesian Age-Period-Cohort (BAPC) model was applied using the R package “bamp,” running 100,000 Markov Chain Monte Carlo iterations with an aging period of 10,000 iterations to refine the predictive accuracy [16]. The data were analyzed and visualized using the R statistical software (version 4.1.2).

## Results

### Worldwide burden in 2021

In 2021, CKD linked to high red meat consumption contributed significantly to global health loss, resulting in approximately 18,652 deaths (95% UI: 0–40,456) and 468,895 DALYs (95% UI: 17–1,027,391). As shown in Table 1, the ASDR was 0.23 per 100,000 population, while the age-standardized DALY rate reached 5.5 per 100,000.(Table 1).

**Table 1:** The number of deaths cases and Age-standardized death rates for chronic kidney disease (CKD) by sex, age, SDI level in 1990 and 2021 and EAPC in Age-standardized death rates from 1990 to 2021

Variable	Number of deaths cases in 1990(95% UI)	The age-standardized deaths rate/100,000 in 1990(95% UI)	Number of deaths cases in 2021(95% UI)	The age-standardized deaths rate/100,000 in 2021(95% UI)	EAPC (95% CI)
Global	5397 (0-12155)	0.16 (0-0.36)	18,652 (0-40456)	0.23 (0-0.5)	1.33 (1.21-1.45)
Sex					
Female	2751 (0-6409)	0.14 (0-0.33)	9439 (0-20963)	0.2 (0-0.45)	1.3 (1.16-1.44)
Male	2646 (0-6073)	0.19 (0-0.43)	9213 (0-20206)	0.26 (0-0.58)	1.25 (1.16-1.35)
Age					
25-29 years	33 (0-98)	0.01 (0-0.02)	72 (0-191)	0.01 (0-0.03)	1.75 (1.65-1.85)
30-34 years	43 (0-126)	0.01 (0-0.03)	97 (0-258)	0.02 (0-0.04)	1.46 (1.37-1.56)
35-39 years	77 (0-203)	0.02 (0-0.06)	159 (0-412)	0.03 (0-0.07)	1.03 (0.91-1.16)
40-44 years	111 (0-304)	0.04 (0-0.11)	265 (0-665)	0.05 (0-0.13)	1.2 (1.1-1.3)
45-49 years	150 (0-389)	0.06 (0-0.17)	447 (0-1059)	0.09 (0-0.22)	1.53 (1.36-1.69)
50-54 years	258 (0-593)	0.12 (0-0.28)	803 (0-1800)	0.18 (0-0.4)	1.52 (1.33-1.72)
55-59 years	380 (0-895)	0.21 (0-0.48)	1197 (0-2763)	0.3 (0-0.7)	1.5 (1.32-1.69)
60-64 years	512 (0-1214)	0.32 (0-0.76)	1460 (0-3372)	0.46 (0-1.05)	1.45 (1.28-1.61)
65-69 years	633 (0-1508)	0.51 (0-1.22)	1954 (0-4366)	0.71 (0-1.58)	1.21 (1.11-1.32)
70-74 years	714 (0-1692)	0.84 (0-2)	2338 (0-5359)	1.14 (0-2.6)	1.06 (1-1.13)
75-79 years	832 (0-1943)	1.35 (0-3.16)	2337 (0-5337)	1.77 (0-4.05)	0.93 (0.87-0.98)
80-84 years	688 (0-1637)	1.95 (0-4.63)	2279 (0-5258)	2.6 (0-6)	1.08 (1.01-1.15)
85-89 years	580 (0-1376)	3.84 (0-9.11)	2421 (0-5767)	5.3 (0-12.61)	1.28 (1.11-1.45)
90-94 years	279 (0-667)	6.51 (0-15.57)	1829 (0-4024)	10.22 (0-22.5)	1.82 (1.61-2.04)
95+ years	106 (0-250)	10.43 (0-24.59)	995 (0-2251)	18.26 (0-41.3)	2.11 (1.91-2.32)
SDI region					
High-middle SDI	1505 (0-3403)	0.18 (0-0.41)	4263 (0-9367)	0.22 (0-0.49)	0.74 (0.61-0.87)
High SDI	1811 (0-4128)	0.17 (0-0.38)	6118 (0-13658)	0.26 (0-0.57)	1.72 (1.59-1.85)
Low-middle SDI	397 (0-926)	0.08 (0-0.18)	1466 (0-3205)	0.11 (0-0.25)	1.28 (1.13-1.42)
Low SDI	201 (0-480)	0.11 (0-0.25)	409 (0-979)	0.1 (0-0.23)	-0.32 (-0.45--0.2)
Middle SDI	1476 (0-3326)	0.17 (0-0.39)	6382 (0-13944)	0.26 (0-0.57)	1.54 (1.38-1.71)

### Demographic distribution

**Fig. 1:**
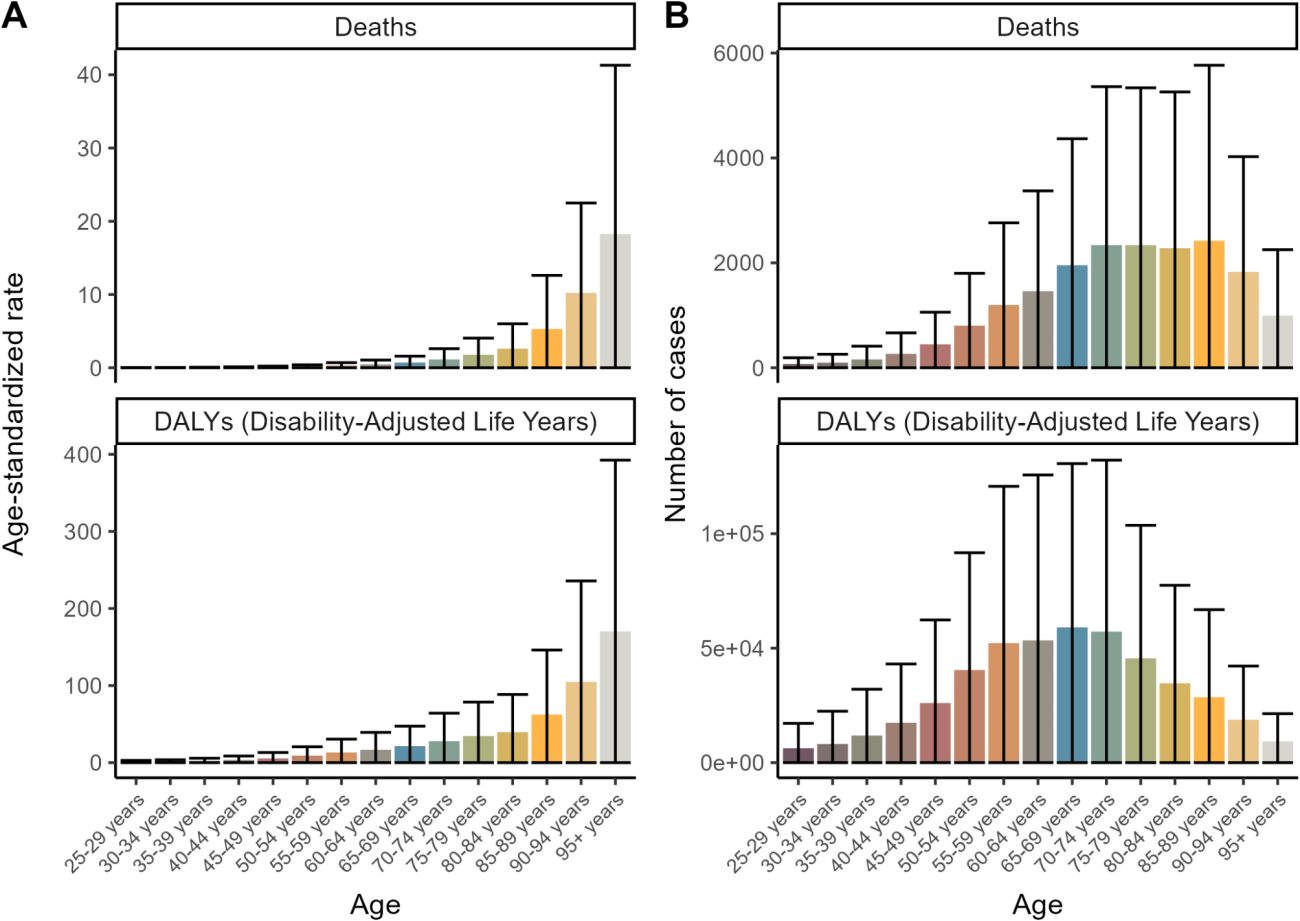
The number of deaths cases, DALYs and age-standardized death, DALYs rates for Global Chronic kidney disease (CKD) by Age in 2021

The burden of CKD associated with high red meat intake showed distinct demographic patterns across age and sex. Figure 1 illustrates the age-dependent distribution, with both mortality and DALY rates generally increasing with age. Mortality peaked in the 85–89 age group, while the highest DALY burden was observed in the 65–69 age group. Notably, the lowest disease burden was found in younger adults (25–29 age group).

**Fig. 2:**
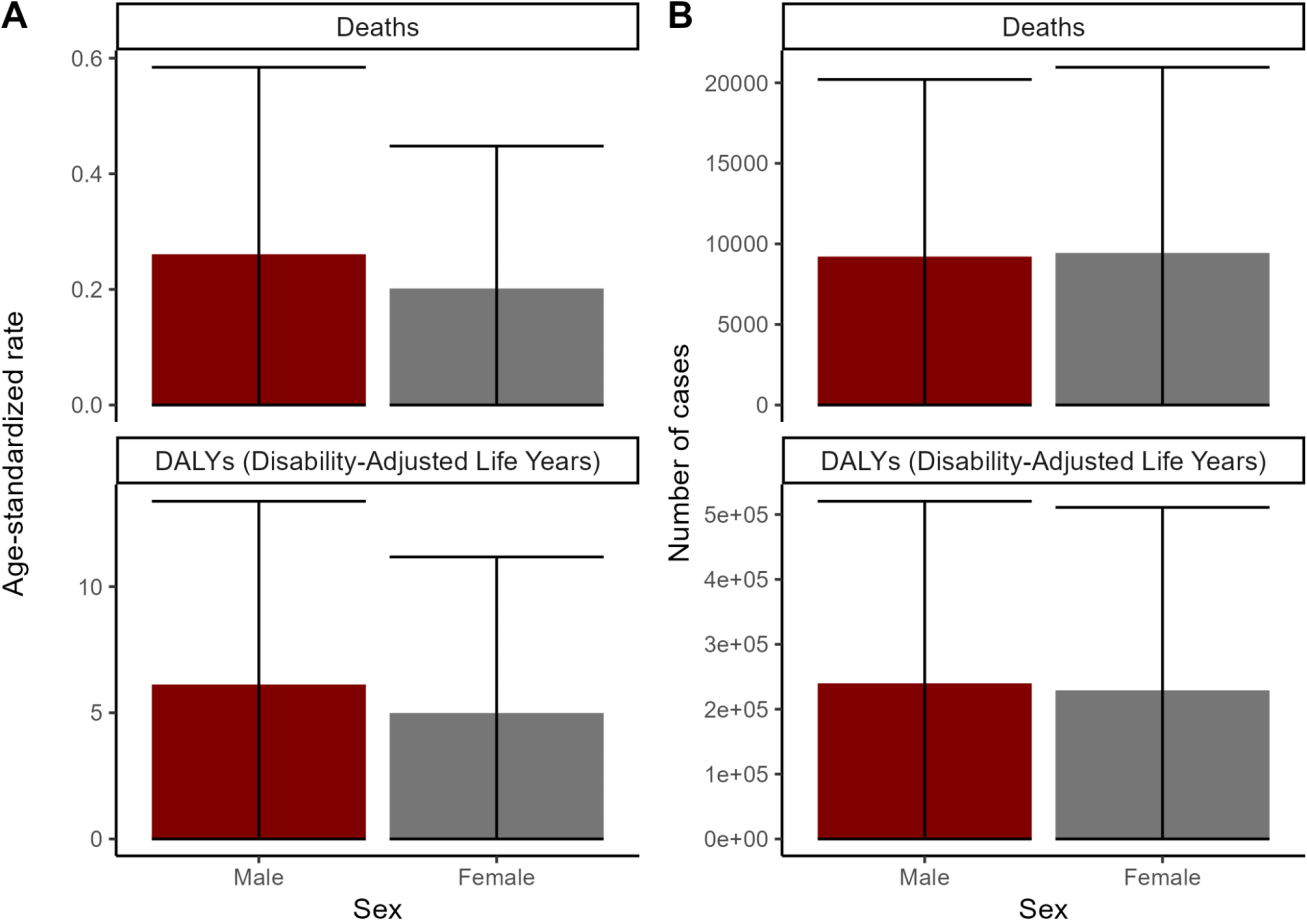
The number of deaths cases, DALYs and age-standardized death, DALYs rates for Global Chronic kidney disease (CKD) by Sex in 2021

Sex-specific analysis revealed that men consistently experienced higher ASDR (0.26 vs. 0.20 per 100,000) and age-standardized DALY rates (6.12 vs. 4.99 per 100,000) compared to women, despite a slightly higher absolute number of deaths among women (Fig. 2).

## Socioeconomic and geographic variations

The burden of CKD linked to high red meat intake varied substantially by socioeconomic development and geographic region. Analysis by Socio-demographic Index (SDI) revealed a gradient pattern, with high and high-middle SDI groups experiencing approximately three times the disease burden of low SDI groups (Figure S1, Tables 1 and 2). The high SDI group had the highest age-standardized DALY rate (6.59 per 100,000), while the low SDI group had the lowest (2.38 per 100,000).

**Table 2:** The number of dalys cases and Age-standardized dalys rates for chronic kidney disease (CKD) by sex, age, SDI level in 1990 and 2021 and EAPC in Age-standardized dalys rates from 1990 to 2021

Variable	Number of DALYs cases in 1990(95% UI)	The age-standardized DALYs rate/100,000 in 1990(95% UI)	Number of DALYs cases in 2021(95% UI)	The age-standardized DALYs rate/100,000 in 2021(95% UI)	EAPC (95% CI)
Global	161,297 (13-360914)	4.2 (0-9.28)	468,895 (17-1027391)	5.5 (0-12.01)	1.07 (0.97-1.17)
Sex					
Female	79,633 (5-178517)	3.82 (0-8.55)	229,040 (10-510972)	4.99 (0-11.18)	1.01 (0.9-1.12)
Male	81,664 (7-185578)	4.72 (0-10.68)	239,855 (9-520423)	6.12 (0-13.38)	1.08 (0.99-1.17)
Age					
25-29 years	3279 (0-9501)	0.74 (0-2.15)	6304 (0-17158)	1.07 (0-2.92)	1.39 (1.3-1.49)
30-34 years	3919 (1-10945)	1.02 (0-2.84)	8145 (0-22457)	1.35 (0-3.72)	1.2 (1.09-1.31)
35-39 years	5972 (2-15311)	1.7 (0-4.35)	11,839 (2-32054)	2.11 (0-5.72)	0.91 (0.82-1)
40-44 years	7664 (1-20738)	2.68 (0-7.24)	17,415 (1-43091)	3.48 (0-8.61)	1.04 (0.94-1.14)
45-49 years	9240 (0-23588)	3.98 (0-10.16)	26,039 (2-62321)	5.5 (0-13.16)	1.33 (1.17-1.48)
50-54 years	13,642 (1-31195)	6.42 (0-14.68)	40,453 (2-91676)	9.09 (0-20.6)	1.37 (1.19-1.55)
55-59 years	17,388 (1-39732)	9.39 (0-21.45)	52,183 (3-120672)	13.19 (0-30.49)	1.34 (1.18-1.51)
60-64 years	20,081 (2-46801)	12.5 (0-29.14)	53,379 (2-125642)	16.68 (0-39.26)	1.25 (1.1-1.4)
65-69 years	20,518 (0-48141)	16.6 (0-38.95)	59,101 (2-130598)	21.43 (0-47.35)	1 (0.92-1.09)
70-74 years	18,543 (1-44164)	21.9 (0-52.17)	57,228 (1-132105)	27.8 (0-64.18)	0.85 (0.8-0.91)
75-79 years	17,551 (0-40135)	28.51 (0-65.2)	45,557 (1-103651)	34.54 (0-78.59)	0.69 (0.64-0.75)
80-84 years	11,513 (0-25948)	32.54 (0-73.35)	34,667 (0-77448)	39.58 (0-88.43)	0.75 (0.69-0.81)
85-89 years	7619 (0-17816)	50.42 (0-117.9)	28,559 (0-66815)	62.46 (0-146.13)	0.89 (0.76-1.02)
90-94 years	3237 (0-7784)	75.55 (0-181.66)	18,749 (0-42174)	104.8 (0-235.75)	1.35 (1.18-1.51)
95+ years	1131 (0-2665)	111.07 (0-261.72)	9277 (0-21389)	170.21 (0-392.43)	1.63 (1.48-1.78)
SDI region					
High-middle SDI	45,069 (5-102888)	4.77 (0-10.75)	105,037 (6-230076)	5.43 (0-11.92)	0.54 (0.44-0.65)
High SDI	51,688 (7-114676)	4.77 (0-10.61)	131,886 (8-282415)	6.59 (0-14.33)	1.3 (1.19-1.41)
Low-middle SDI	12,206 (0-28832)	2.02 (0-4.74)	44,233 (1-98204)	2.99 (0-6.62)	1.36 (1.22-1.51)
Low SDI	6015 (0-14492)	2.63 (0-6.3)	12,706 (0-30951)	2.38 (0-5.66)	-0.37 (-0.47--0.27)
Middle SDI	46,113 (1-103190)	4.42 (0-9.85)	174,670 (3-384704)	6.5 (0-14.21)	1.52 (1.33-1.7)

**Fig. 3:**
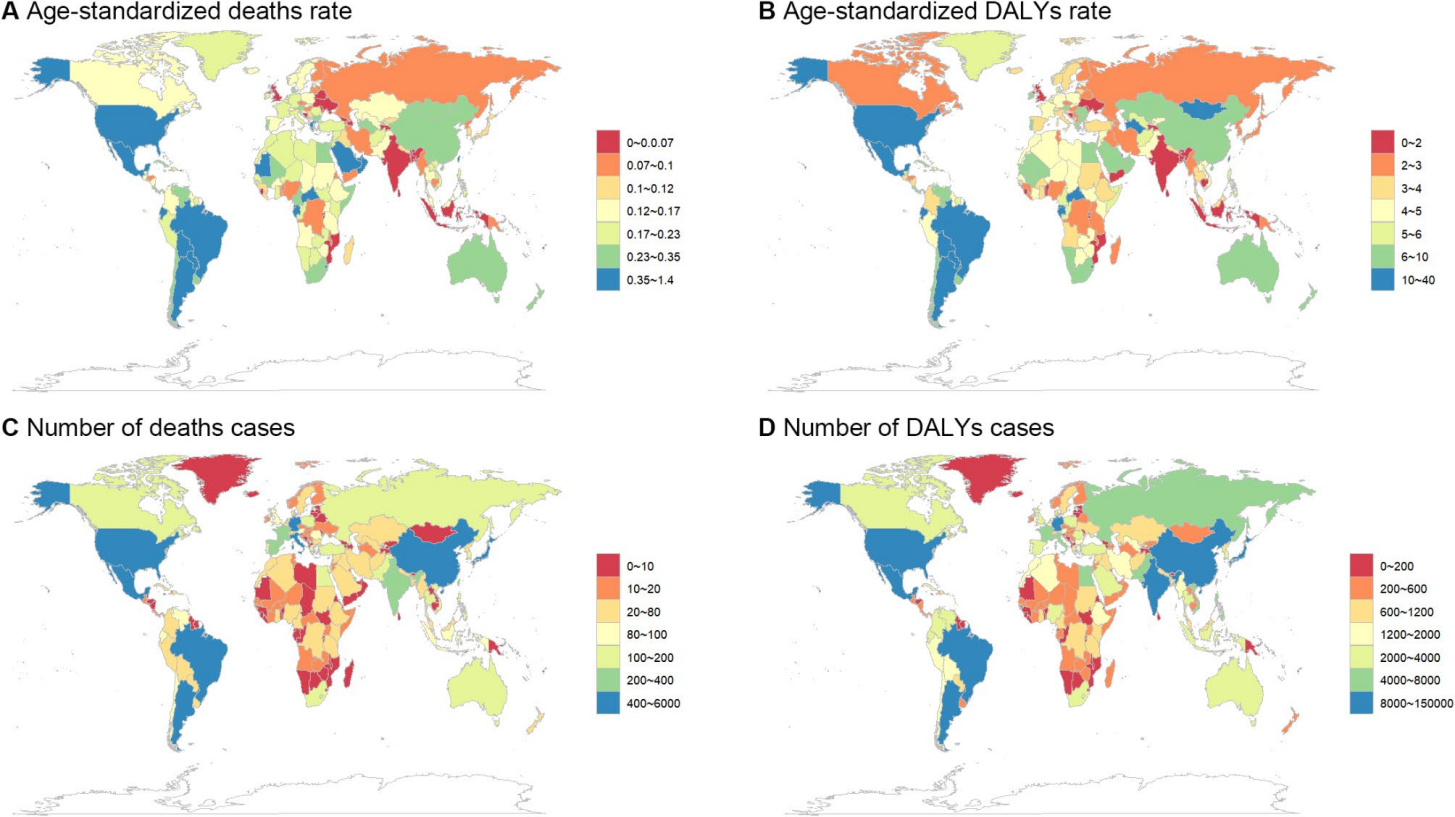
The number of deaths cases, DALYs and age-standardized death, DALYs rates for Global Chronic kidney disease (CKD) by countries in 2021

Significant geographic variations existed across GBD regions and countries (Figs. 3 and S2). Tropical Latin America experienced the highest burden (ASDR: 0.59 per 100,000; DALY rate: 15.82 per 100,000), while Southeast Asia had the lowest (ASDR: 0.04 per 100,000; DALY rate: 1.07 per 100,000). At the country level, the highest mortality rates were observed in American Samoa, Gabon, and Fiji, while Sri Lanka, Ukraine, and Tajikistan had the lowest rates.

## Temporal trends from 1990 to 2021

### Global trends

**Fig. 4:**
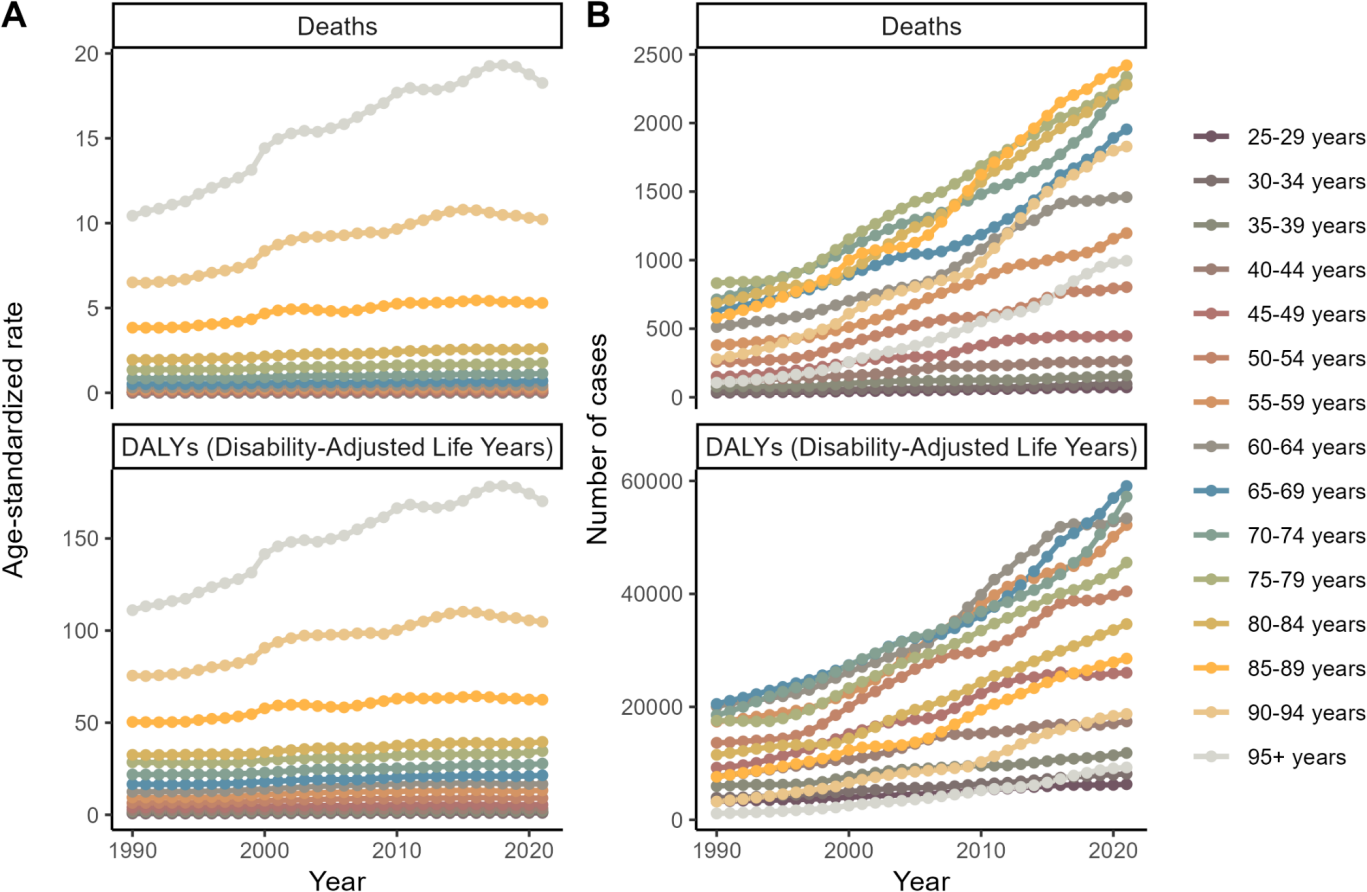
Trends of the number of deaths cases, DALYs and age-standardized death, DALYs rates for Global Chronic kidney disease (CKD) by Age from 1990 to 2021

**Fig. 5:**
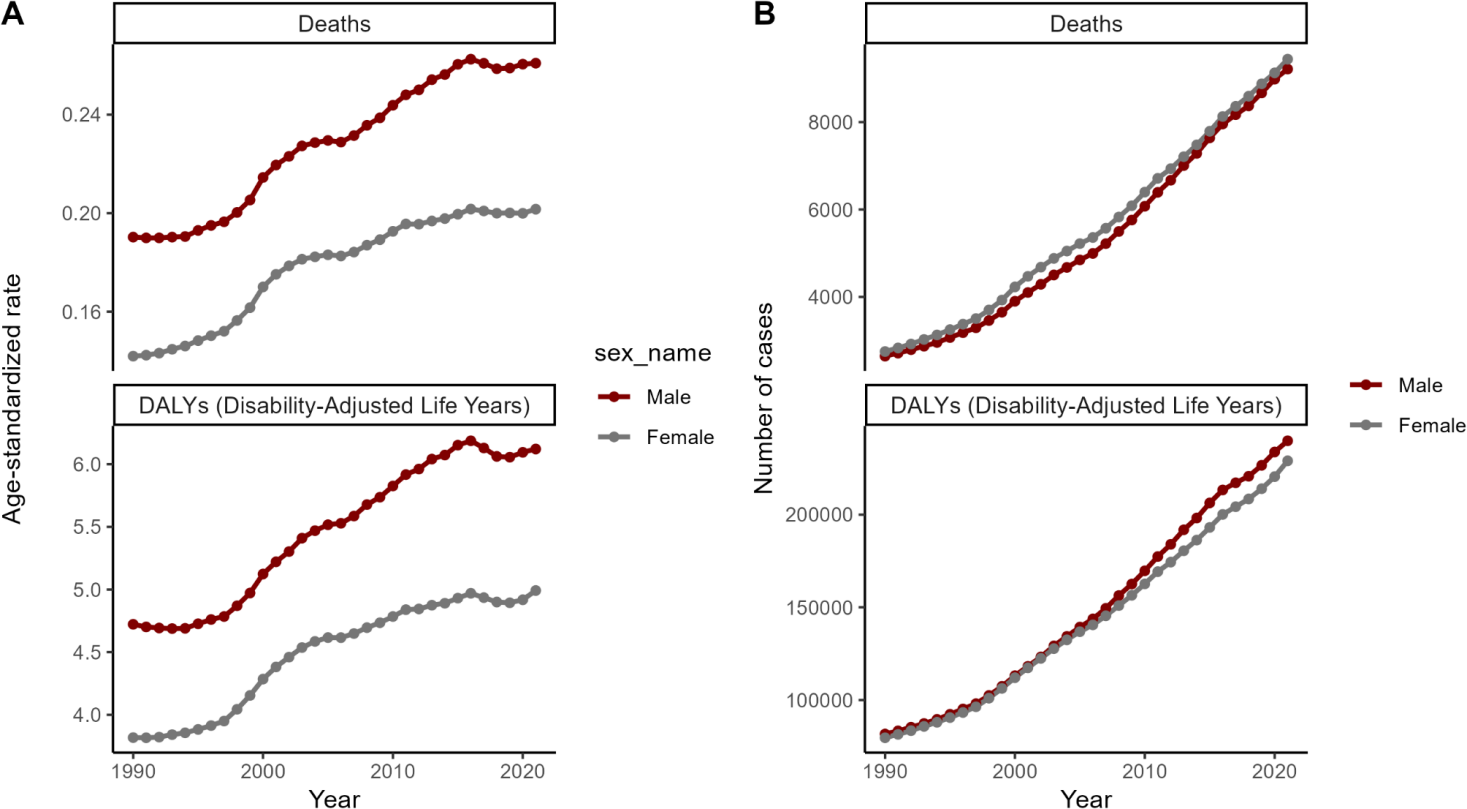
Trends of the number of deaths cases, DALYs and age-standardized death, DALYs rates for Global Chronic kidney disease (CKD) by Sex from 1990 to 2021

The temporal analysis revealed several important patterns:

1. All age groups showed an increasing burden over time, with the most pronounced increases in the 65–84 age group (Fig. 4). However, individuals aged 95 + experienced a notable decline in both mortality and DALY rates from 2018 to 2021.

2. The male-female disparity persisted throughout the study period, with men consistently experiencing higher disease burden than women (Fig. 5). Both sexes showed a similar pattern of increase, with a temporary decline observed between 2016 and 2018.

3. Analysis by SDI groups showed that middle and high SDI groups experienced the largest increases in disease burden over time, while low SDI regions actually showed a declining trend (EAPC: -0.32) (Figure S4).

### Regional and Country-Level trends

**Fig. 6:**
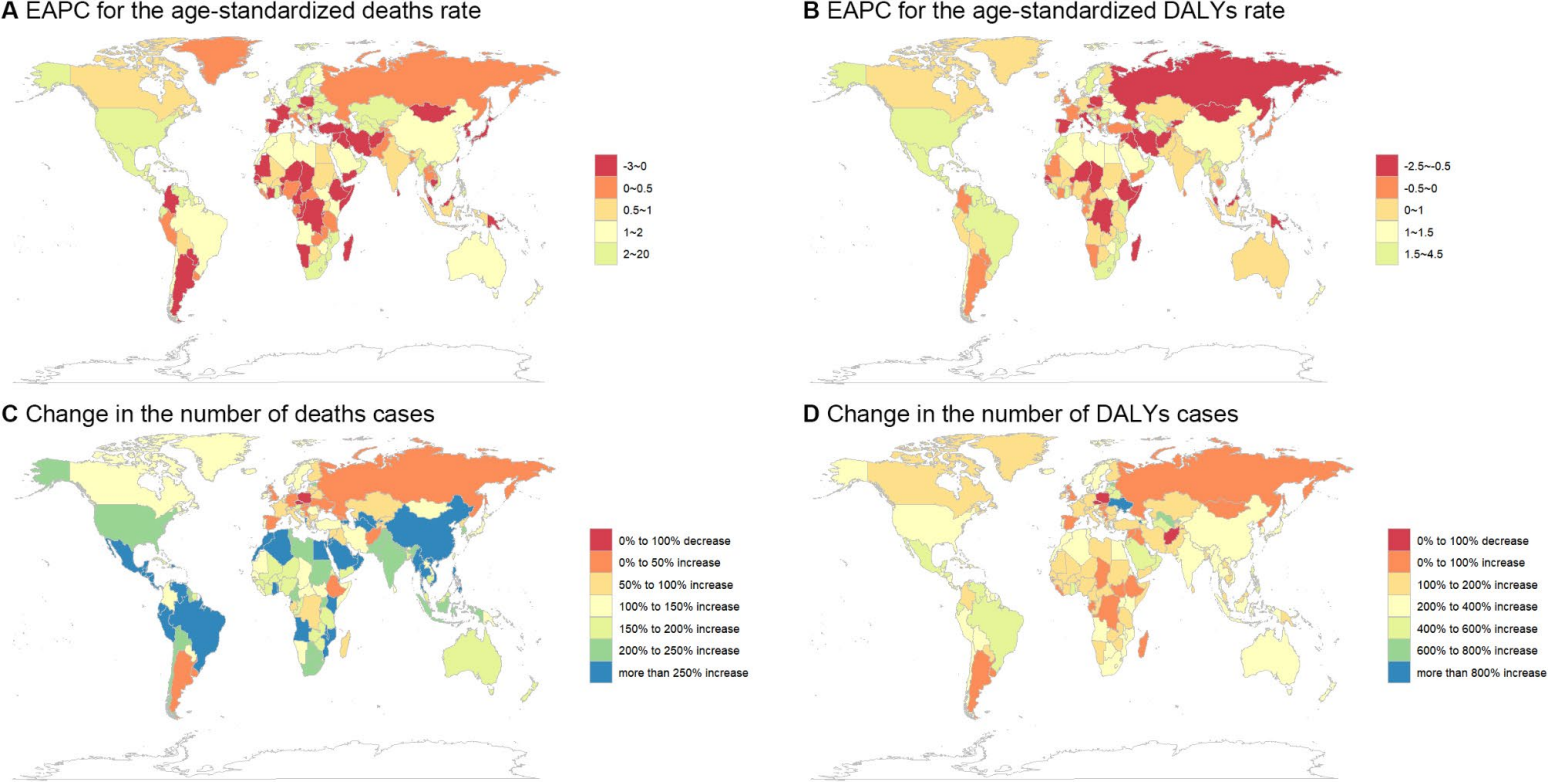
Trends of the number of deaths cases, DALYs and age-standardized death, DALYs rates for Global Chronic kidney disease (CKD) by countries from 1990 to 2021

Geographic trend analysis revealed substantial heterogeneity (Figs. 6 and S5). Of the 54 GBD regions, eight showed declining ASDR, including Central Sub-Saharan Africa (EAPC: -0.81), while 46 regions showed increases, with the largest rises in high-income North America (EAPC: 2.96) and Central Asia (EAPC: 2.61).

At the country level, 149 countries experienced increasing ASDR, while 54 showed decreases. The most rapid increases occurred in Armenia, Estonia, and Latvia, while the most substantial decreases were observed in Poland, Cyprus, and the Czech Republic. Similar patterns were observed for DALY rates, with 142 countries showing increases and 62 showing decreases.

### Future projections (2022–2046)

We employed two complementary forecasting methodologies to project future trends in CKD burden associated with high red meat intake: the APC model and the BAPC model. These models provide different approaches to account for temporal effects and uncertainty in long-term forecasting.

### Comparative projection results

The APC and BAPC models yielded notably different projections for the 25-year forecast period:

### Male burden projections

Under the APC model, male ASDR is projected to remain relatively stable (0.26–0.27 per 100,000) with male age-standardized DALY rates showing a modest decrease from 6.15 to 6.04 per 100,000 (Figure S6).

In contrast, the BAPC model projects stable male ASDR (approximately 0.26 per 100,000) but predicts an increasing trend in age-standardized DALY rates from 6.18 to 6.61 per 100,000 (Figure S7).

### Female burden projections

The APC model suggests a slight decrease in female ASDR (0.20 to 0.19 per 100,000) and age-standardize DALY rates (4.97 to 4.90 per 100,000).

Conversely, the BAPC model projects an increasing trend in female burden, with ASDR rising from 0.20 to 0.22 per 100,000 and a more substantial increase in age-standardize DALY rates that is projected to surpass male age-standardize DALY rates by 2045.

These divergent projections highlight the inherent uncertainty in forecasting disease burden and suggest that different methodological approaches may capture distinct aspects of emerging trends in red meat consumption and its health impacts.

## Discussion

### Main findings

This study presents the first comprehensive global analysis of CKD burden attributable to high red meat intake using the GBD 2021 dataset. Our key findings reveal four critical patterns: First, there has been a significant increase in the global burden of CKD linked to high red meat consumption between 1990 and 2021, with ASDR and age-standardized DALY rates rising by 1.33% and 1.07% annually, respectively. Second, notable demographic disparities exist, with males consistently experiencing higher burden than females, and mortality peaking in the 85–89 age group. Third, substantial geographic and socioeconomic variations were observed, with high SDI groups experiencing nearly three times the burden of low SDI groups (age-standardized DALY rate: 6.59 vs. 2.38 per 100,000), and Latin America showing particularly elevated rates. Fourth, despite the overall increasing trend, we identified several important temporal anomalies, including a temporary decline between 2016 and 2018 and decreasing rates among individuals over 95 years of age between 2018 and 2021.

### Gender disparities in CKD burden

The persistent gender disparity in CKD burden can be attributed to both biological and sociocultural factors. Biologically, androgens, particularly testosterone, enhance kidney sensitivity to specific red meat components through receptor-mediated gene expression in renal tissue [17]. Socioculturally, traditional gender norms link meat consumption with masculinity and strength, leading to higher intake among males [18]. Even when aware of health risks, men are generally less likely to proactively reduce red meat consumption [19].

### Temporal trends and contributing factors

Within the overall rising trend, a temporary decline in ASDR and age-standardized DALY rates occurred between 2016 and 2018 for both sexes. Several factors likely contributed to this phenomenon. First, the increasing popularity of plant-based diets and heightened public awareness of diet-related chronic disease prevention may have led to reduced red meat consumption. Second, stricter dietary guidelines and health education campaigns in multiple countries provided structured guidance for behavior change [20]. Additionally, the rapid growth of the alternative protein market expanded health-conscious dietary options [21]. While this decline was temporary, it offers valuable insights into the potential impact of dietary interventions and informs future prevention strategies.

The burden of disease generally increases with age, peaking in the 85–89 age group. This trend indicates a cumulative impact of red meat intake on kidney function over time [22]. The natural decline in renal function with aging, coupled with comorbidities such as hypertension and diabetes, likely amplifies red meat’s detrimental effects [7, 23]. However, among individuals over 95 years old, a notable decline in disease burden was observed between 2018 and 2021. Possible explanations include improved healthcare awareness, better dietary management by caregivers, and selective survival effects in this age group. Moreover, the COVID-19 pandemic and related social isolation may have contributed by limiting processed red meat consumption among the elderly [24].

### Socioeconomic and regional variations

High and high-middle SDI groups bear a disproportionately higher CKD burden, reflecting a combination of socioeconomic and cultural factors. Increased red meat accessibility due to well-developed cold chain logistics, dietary shifts in urbanized areas, and differences in healthcare system capacities for disease detection and reporting all contribute to this disparity [25], Notably, Latin America exhibits a particularly high burden, potentially linked to its unique dietary culture and traditional meat processing methods [26].

### Pathophysiological mechanisms

Red meat consumption contributes to kidney dysfunction through multiple interconnected mechanisms. First, heme iron induces oxidative stress and inflammatory responses, directly damaging renal tubular epithelial cells [27, 28]. Second, saturated fatty acids in red meat activate inflammatory signaling pathways and induce lipotoxicity, impairing renal function and exacerbating atherosclerosis, which compromises renal microvascular health [29]. Additionally, the high protein and purine content in red meat increases metabolic strain on the kidneys, while additives in processed meats may exert nephrotoxic effects. These mechanisms interact in a complex network, where oxidative stress amplifies lipotoxic effects and inflammation accelerates multiple pathogenic pathways [30]. Genetic predispositions and environmental factors further modulate these processes, contributing to population-level variations in disease susceptibility.

#### Comorbidities and disease interactions

CKD linked to high red meat intake is closely associated with other chronic conditions, particularly cardiovascular disease (CVD) and type 2 diabetes (T2D) [31]. Excessive red meat consumption has been implicated in atherosclerosis, forming a pathological cascade from vascular damage to renal microcirculatory dysfunction [25]. Furthermore, the co-occurrence of these chronic conditions often leads to a vicious cycle, where one disease exacerbates another, accelerating kidney damage [32].Furthermore, the co-occurrence of these chronic conditions often leads to a vicious cycle, where one disease exacerbates another, accelerating kidney damage.

### Implications for intervention and prevention

Reducing the CKD burden linked to high red meat intake requires multi-level, systematic interventions. First, in clinical practice, early screening and intervention systems for high-risk populations are essential. These should include not only kidney function monitoring but also dietary assessments focusing on red meat intake. Additionally, tailored nutritional guidance for the elderly is crucial, given their comorbidities and dietary needs [33, 34]. Second, from a public health perspective, region-specific prevention strategies are vital. These include implementing stricter regulations on processed red meat safety, formulating intake recommendations aligned with local dietary habits, and launching targeted health education campaigns. Additionally, integrating these efforts into broader chronic disease prevention frameworks can enhance overall effectiveness [35].Finally, strengthening multi-sectoral collaboration is essential for effective policy implementation. Coordinated efforts among governments, healthcare systems, and communities can optimize intervention outcomes. Furthermore, pooling resources will ensure the sustainability of these measures, while regular evaluations will help assess effectiveness and enable timely adjustments [36].

#### Strengths and limitations

Strengths of this study include the application of standardized GBD methodology providing consistent and comparable estimates across regions and time periods, extensive global coverage encompassing 204 countries and territories, and a long-term trend analysis spanning over three decades that enables the identification of both persistent patterns and temporal anomalies. However, several limitations should be acknowledged. The attribution of CKD burden to red meat intake relies on epidemiological data quality, which varies across populations. Uncertainty intervals in our estimates, with lower bounds approaching zero in some cases, highlight data limitations and regional heterogeneity. Moreover, our analysis is based on ecological correlations and cannot establish causality or account for individual variations in dietary habits and genetic susceptibility. Although the GBD methodology is robust, it may not fully account for confounding factors like food preparation methods and concurrent dietary patterns. Additionally, the COVID-19 pandemic likely influenced healthcare access and dietary behaviors, potentially affecting recent trends [37]. Finally, predictive models are inherently uncertain, as they assume historical patterns will persist despite evolving dietary trends, food systems, and healthcare advancements. Despite these limitations, our findings provide valuable insights for developing targeted, region-specific prevention strategies.

## Conclusion

Our comprehensive analysis of GBD data reveals that the global burden of CKD attributable to high red meat intake has increased significantly from 1990 to 2021, with ASDR and age-standardized DALY rates rising by 1.33% and 1.07% annually, respectively. This burden is unevenly distributed across demographic and geographic populations. Males consistently experience higher disease rates than females, while mortality peaks in the 85–89 age group. Additionally, high-SDI groups bear nearly three times the burden of low-SDI groups. Given these disparities, dietary guidance should be prioritized, particularly for populations with high red meat consumption. Future research should focus on two critical areas: (1) further elucidating the biological mechanisms through which red meat consumption contributes to kidney dysfunction, and (2) developing and evaluating prevention strategies.

## Electronic supplementary material

Below is the link to the electronic supplementary material.

Supplementary Material 1

Supplementary Material 2

Supplementary Material 3

Supplementary Material 4

Supplementary Material 5

Supplementary Material 6

Supplementary Material 7

## Data Availability

The datasets supporting the conclusions of this article are available in the Global Health Data Exchange repository at https://ghdx.healthdata.org/gbd-2021/sources.

## Abbreviations

BMI: Body Mass Index
CKD: Chronic Kidney Disease
CVD: Cardiovascular Disease
DALY: Disability-Adjusted Life Year
GBD: Global Burden of Disease
HDI: Human Development Index
ICD: International Classification of Diseases
IHME: Institute for Health Metrics and Evaluation
SDI: Socio-demographic Index
YLD: Years Lived with Disability
ASDR: Age-standardized Deaths Rate

## References

[1] Morales Palomares S, Parozzi M, Ferrara G, Andreoli D, Godino L, Gazineo D, Anastasi G, Sguanci M, Mancin S. Olfactory dysfunctions and chronic kidney disease: A scoping review. J Ren Nutrition: Official J Council Ren Nutr Natl Kidney Foundation 2024;35(1):4–14. 10.1053/j.jrn.2024.06.007 38925323

[2] Ali J, Shah S, Nadeem M, Mahmood A, Ahmad U. A comparative study of the epidemiology and risk factors of chronic kidney disease among rural and urban residents in Peshawar, Pakistan. Cureus. 2024;16(7):e64215. 39131032 10.7759/cureus.64215 PMC11310797

[3] Harambat J, Ekulu PM. Inequalities in access to pediatric ESRD care: a global health challenge. Pediatr Nephrol. 2016;31(3):353–8. 26628281 10.1007/s00467-015-3263-7

[4] Hossain MP, Goyder EC, Rigby JE, El Nahas M. CKD and poverty: a growing global challenge. Am J Kidney Diseases: Official J Natl Kidney Foundation. 2009;53(1):166–74. 10.1053/j.ajkd.2007.10.047 19101400

[5] Ong KL, Marklund M, Huang L, Rye KA, Hui N, Pan XF, Rebholz CM, Kim H, Steffen LM, van Westing AC, et al. Association of Omega 3 polyunsaturated fatty acids with incident chronic kidney disease: pooled analysis of 19 cohorts. BMJ (Clinical Res ed). 2023;380:e072909. 10.1136/bmj-2022-072909 PMC9846698 36653033

[6] Banerjee T, Carrero JJ, McCulloch C, Burrows NR, Siegel KR, Morgenstern H, Saran R, Powe NR. Dietary factors and prevention: risk of End-Stage kidney disease by fruit and vegetable consumption. Am J Nephrol. 2021;52(5):356–67. 34044392 10.1159/000514754 PMC8263504

[7] Lew QJ, Jafar TH, Koh HW, Jin A, Chow KY, Yuan JM, Koh WP. Red meat intake and risk of ESRD. J Am Soc Nephrology: JASN. 2017;28(1):304–12. 10.1681/ASN.2016030248 PMC5198288 27416946

[8] Mikhailova NA. [The value of a low-protein diet and ketoanalogues of essential amino acids in the сontrol of protein carbamylation and toxic effects of Urea in chronic kidney disease]. Ter Arkh. 2021;93(6):729–35. 10.26442/00403660.2021.06.200915 36286841

[9] Murray CJL. Findings from the global burden of disease study 2021. Lancet (London England). 2024;403(10440):2259–62. 38762327 10.1016/S0140-6736(24)00769-4

[10] Global burden and strength of evidence for. 88 Risk factors in 204 countries and 811 subnational locations, 1990–2021: a systematic analysis for the global burden of disease study 2021. Lancet (London England). 2024;403(10440):2162–203. 38762324 10.1016/S0140-6736(24)00933-4 PMC11120204

[11] Sergl HG. [The psychology of sucking habits]. Fortschr Kieferorthop. 1985;46(2):101–12. 3858209 10.1007/BF02167524

[12] González N, Marquès M, Nadal M, Domingo JL. Meat consumption: which are the current global risks? A review of recent (2010–2020) evidences. Food Res Int (Ottawa Ont). 2020;137:109341. 10.1016/j.foodres.2020.109341 PMC7256495 33233049

[13] Yang X, Quam MBM, Zhang T, Sang S. Global burden for dengue and the evolving pattern in the past 30 years. J Travel Med 2021, 28(8). 10.1093/jtm/taab146 34510205

[14] Cao G, Liu J, Liu M. Global, regional, and National incidence and mortality of neonatal preterm birth, 1990–2019. JAMA Pediatr. 2022;176(8):787–96. 35639401 10.1001/jamapediatrics.2022.1622 PMC9157382

[15] Chen J, Qiu Y, Wu W, Yang R, Li L, Yang Y, Yang X, Xu L. Trends and projection of the incidence of active pulmonary tuberculosis in Southwestern China: Age-Period-Cohort analysis. JMIR Public Health Surveillance. 2023;9:e48015. 38157236 10.2196/48015 PMC10787335

[16] Hu W, Yang J. Effect of ambient Ozone pollution on disease burden globally: A systematic analysis for the global burden of disease study 2019. Sci Total Environ. 2024;926:171739. 38508259 10.1016/j.scitotenv.2024.171739

[17] Tan SJ, Hu JP, Zhong Y. The effects of androgen on sodium excretion and the renin-angiotensin system in high salt-induced hypertensive male rats. Saudi Med J. 2009;30(2):305–7. 19198728

[18] Camilleri L, Kirkovski M, Scarfo J, Jago A, Gill PR. Understanding the meat-Masculinity link: traditional and Non-Traditional masculine norms predicting Men’s meat consumption. Ecol Food Nutr. 2024;63(4):355–86. 38835162 10.1080/03670244.2024.2361818

[19] Frąckiewicz J, Sawejko Z, Ciecierska A, Drywień ME. Gender as a factor influencing the frequency of meat and fish consumption in young adults. Rocz Panstw Zakl Hig. 2023;74(4):373–84. 38116797 10.32394/rpzh.2023.0276

[20] Johnston BC, Zeraatkar D, Han MA, Vernooij RWM, Valli C, El Dib R, Marshall C, Stover PJ, Fairweather-Taitt S, Wójcik G, et al. Unprocessed red meat and processed meat consumption: dietary guideline recommendations from the nutritional recommendations (NutriRECS) consortium. Ann Intern Med. 2019;171(10):756–64. 31569235 10.7326/M19-1621

[21] Wang Y, Tibbetts SM, McGinn PJ. Microalgae as sources of High-Quality protein for human food and protein supplements. Foods 2021, 10(12). 10.3390/foods10123002 PMC8700990 34945551

[22] Kamper AL, Strandgaard S. Long-Term effects of High-Protein diets on renal function. Annu Rev Nutr. 2017;37:347–69. 28637384 10.1146/annurev-nutr-071714-034426

[23] Lu TY, Zhang WS, Zhu T, Jiang CQ, Zhu F, Jin YL, Lam TH, Cheng KK, Xu L. Associations of meat, fish and seafood consumption with kidney function in middle-aged to older Chinese: a cross-sectional study based on the Guangzhou biobank cohort study. BMJ Open. 2023;13(10):e073738. 37802614 10.1136/bmjopen-2023-073738 PMC10565302

[24] Ferrante G, Camussi E, Piccinelli C, Senore C, Armaroli P, Ortale A, Garena F, Giordano L. Did social isolation during the SARS-CoV-2 epidemic have an impact on the lifestyles of citizens? Epidemiol Prev. 2020;44(5–6 Suppl 2):353–62. 33412829 10.19191/EP20.5-6.S2.137

[25] Frank SM, Jaacks LM, Batis C, Vanderlee L, Taillie LS. Patterns of red and processed meat consumption across North America: A nationally representative Cross-Sectional comparison of dietary recalls from Canada, Mexico, and the united States. Int J Environ Res Public Health 2021, 18(1). 10.3390/ijerph18010357 PMC7796493 33466518

[26] Santin F, Canella DS, Avesani CM. Food consumption in chronic kidney disease: association with sociodemographic and geographical variables and comparison with healthy individuals. J Ren Nutr. 2019;29(4):333–42. 30591359 10.1053/j.jrn.2018.10.010

[27] Zager RA, Johnson AC. Progressive histone alterations and Proinflammatory gene activation: consequences of Heme protein/iron-mediated proximal tubule injury. Am J Physiol Ren Physiol. 2010;298(3):F827–837. 10.1152/ajprenal.00683.2009 PMC2838607 20032114

[28] Martines AM, Masereeuw R, Tjalsma H, Hoenderop JG, Wetzels JF, Swinkels DW. Iron metabolism in the pathogenesis of iron-induced kidney injury. Nat Rev Nephrol. 2013;9(7):385–98. 23670084 10.1038/nrneph.2013.98

[29] Noels H, Lehrke M, Vanholder R, Jankowski J. Lipoproteins and fatty acids in chronic kidney disease: molecular and metabolic alterations. Nat Rev Nephrol. 2021;17(8):528–42. 33972752 10.1038/s41581-021-00423-5

[30] Jung SW, Kim SM, Kim YG, Lee SH, Moon JY. Uric acid and inflammation in kidney disease. Am J Physiol Ren Physiol. 2020;318(6):F1327–40. 10.1152/ajprenal.00272.2019 32223310

[31] Li G, Jiang J, Li Z. The relationship between processed meat, red meat, and risk of cardiovascular disease and type 2 diabetes: A Mendelian randomization study. Eur J Prev Cardiol 2024;00:1–8. 10.1093/eurjpc/zwae117 38525976

[32] Ghosh SS, Righi S, Krieg R, Kang L, Carl D, Wang J, Massey HD, Sica DA, Gehr TW, Ghosh S. High fat high cholesterol diet (Western diet) aggravates atherosclerosis, hyperglycemia and renal failure in nephrectomized LDL receptor knockout mice: role of intestine derived lipopolysaccharide. PLoS ONE. 2015;10(11):e0141109. 26580567 10.1371/journal.pone.0141109 PMC4651339

[33] Johnson DW, Atai E, Chan M, Phoon RK, Scott C, Toussaint ND, Turner GL, Usherwood T, Wiggins KJ. KHA-CARI guideline: early chronic kidney disease: detection, prevention and management. Nephrol (Carlton). 2013;18(5):340–50. 10.1111/nep.12052 23506545

[34] Windahl K, Chesnaye NC, Irving GF, Stenvinkel P, Almquist T, Lidén MK, Drechsler C, Szymczak M, Krajewska M, de Rooij E, et al. The safety of a low-protein diet in older adults with advanced chronic kidney disease. Nephrol Dial Transpl. 2024;39(11):1867–75. 10.1093/ndt/gfae077 PMC11648958 38544335

[35] Wolk A. Potential health hazards of eating red meat. J Intern Med. 2017;281(2):106–22. 27597529 10.1111/joim.12543

[36] Mikkelsen BE, Novotny R, Gittelsohn J. Multi-Level, Multi-Component approaches to community based interventions for healthy Living-A three case comparison. Int J Environ Res Public Health 2016, 13(10). 10.3390/ijerph13101023 PMC5086762 27775630

[37] Zupo R, Castellana F, Sardone R, Sila A, Giagulli VA, Triggiani V, Cincione RI, Giannelli G, De Pergola G. Preliminary Trajectories in Dietary Behaviors during the COVID-19 Pandemic: A Public Health Call to Action to Face Obesity. *International journal of environmental research and public health* 2020, 17(19). 10.3390/ijerph17197073 PMC7579065 32992623

