# Supplementary figures and images for: Trends in the burden of chronic kidney disease related to high red meat intake from 1990 to 2021
Source: BMC Public Health. 2025 Apr 8;25:1319. doi: 10.1186/s12889-025-22560-3 (PMC11978116; doi:10.1186/s12889-025-22560-3)

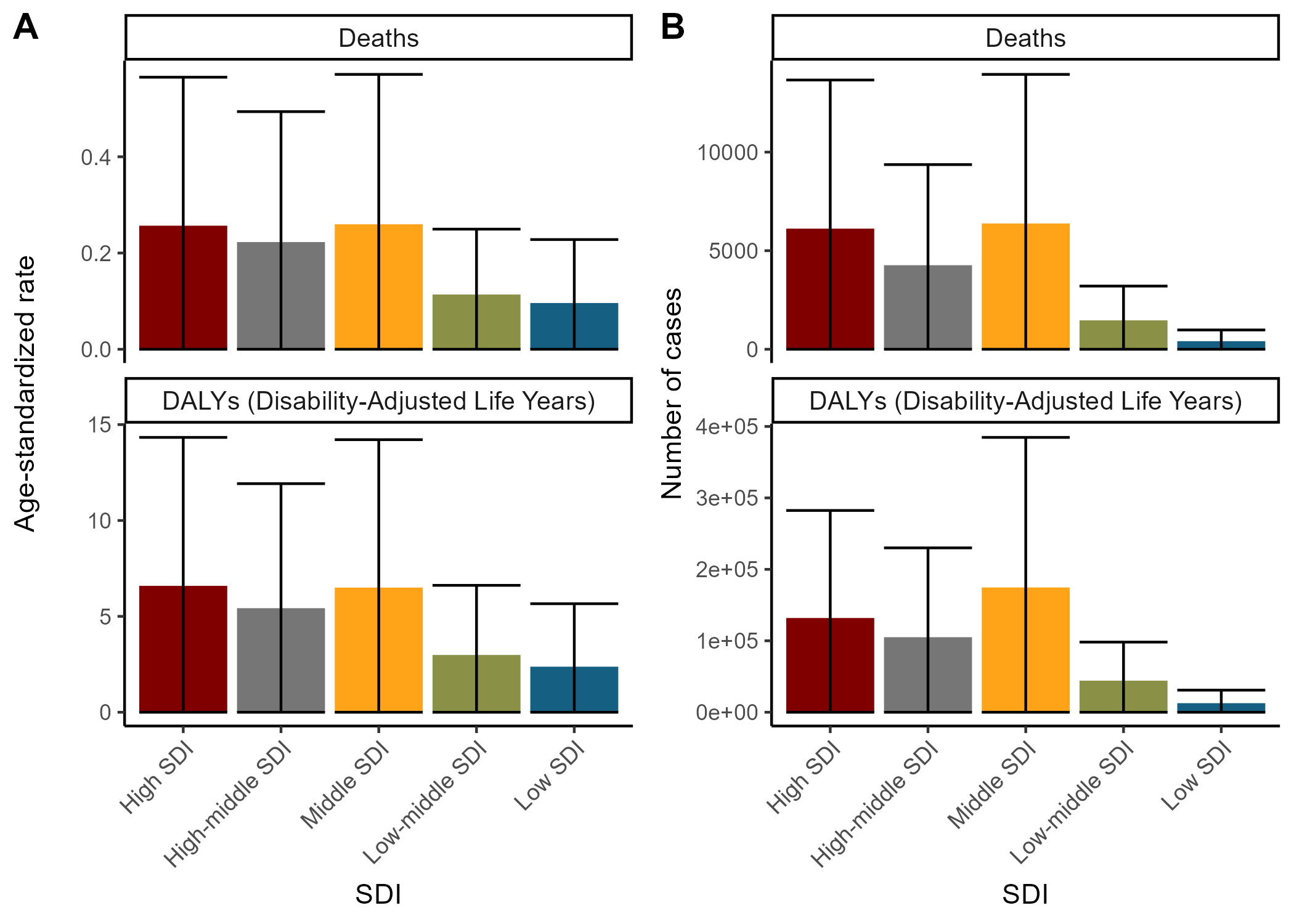

Supplement: Supplementary file 1 — Supplementary Material 1 [file 12889_2025_22560_MOESM1_ESM.png]

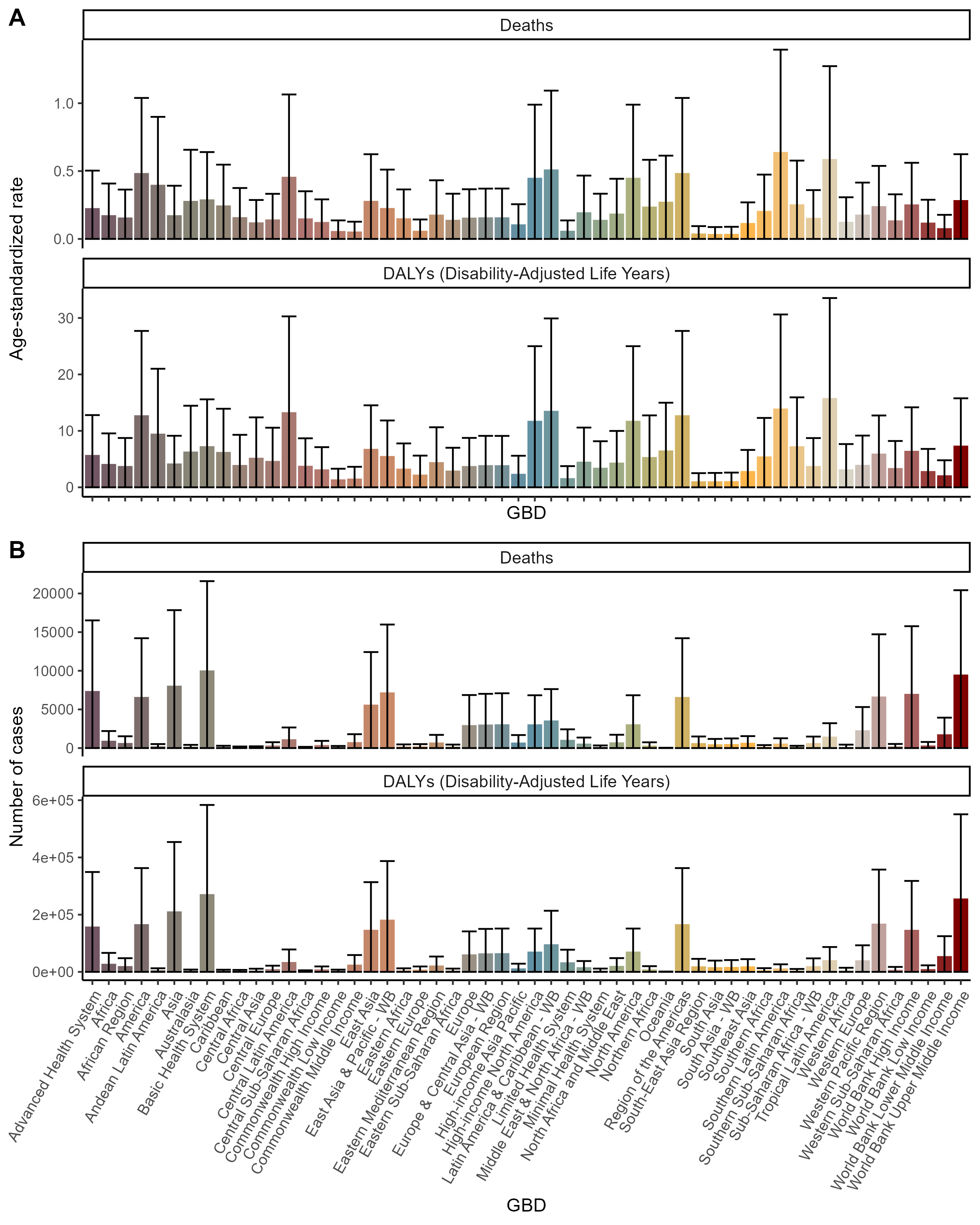

Supplement: Supplementary file 2 — Supplementary Material 2 [file 12889_2025_22560_MOESM2_ESM.png]

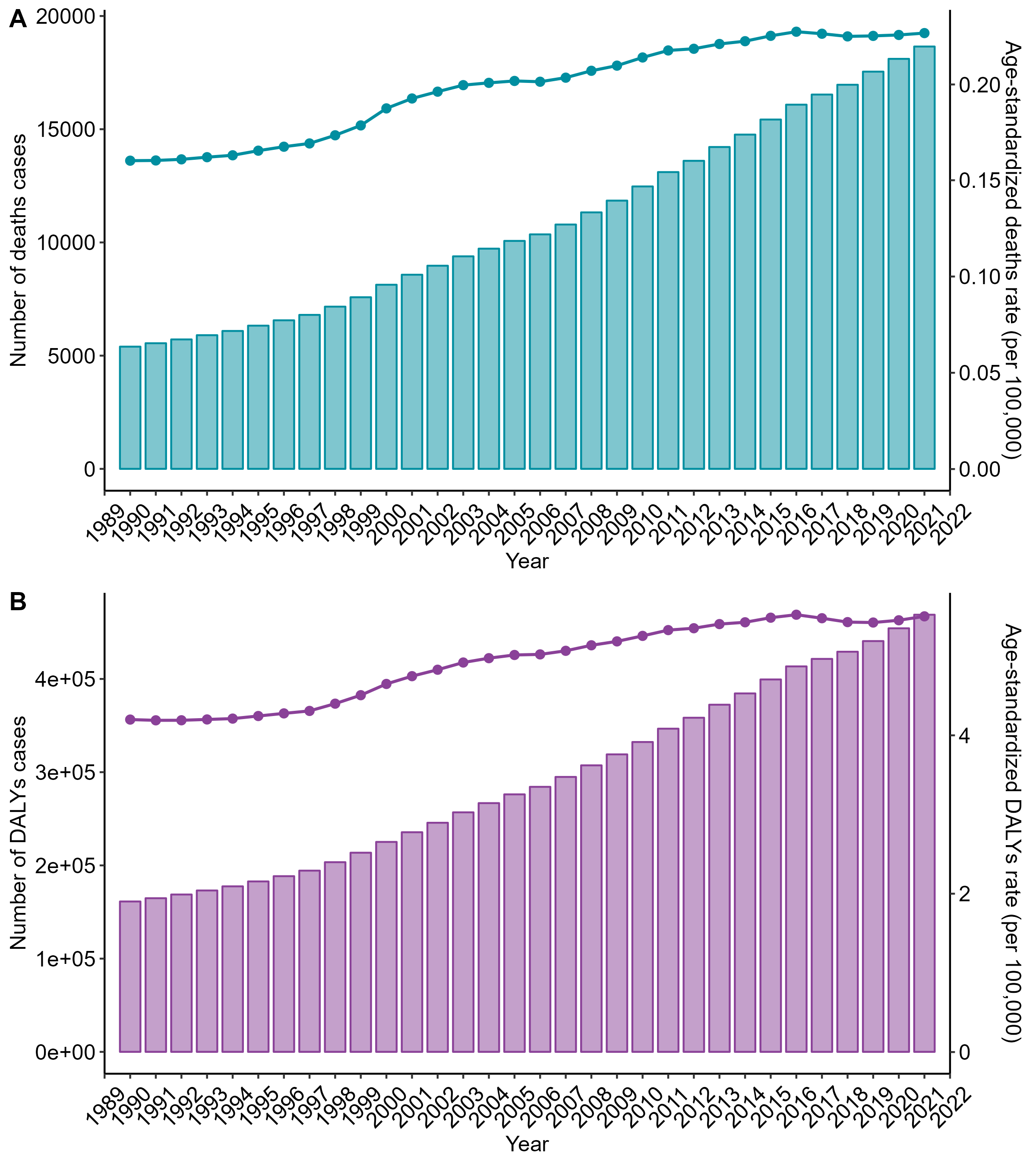

Supplement: Supplementary file 3 — Supplementary Material 3 [file 12889_2025_22560_MOESM3_ESM.png]

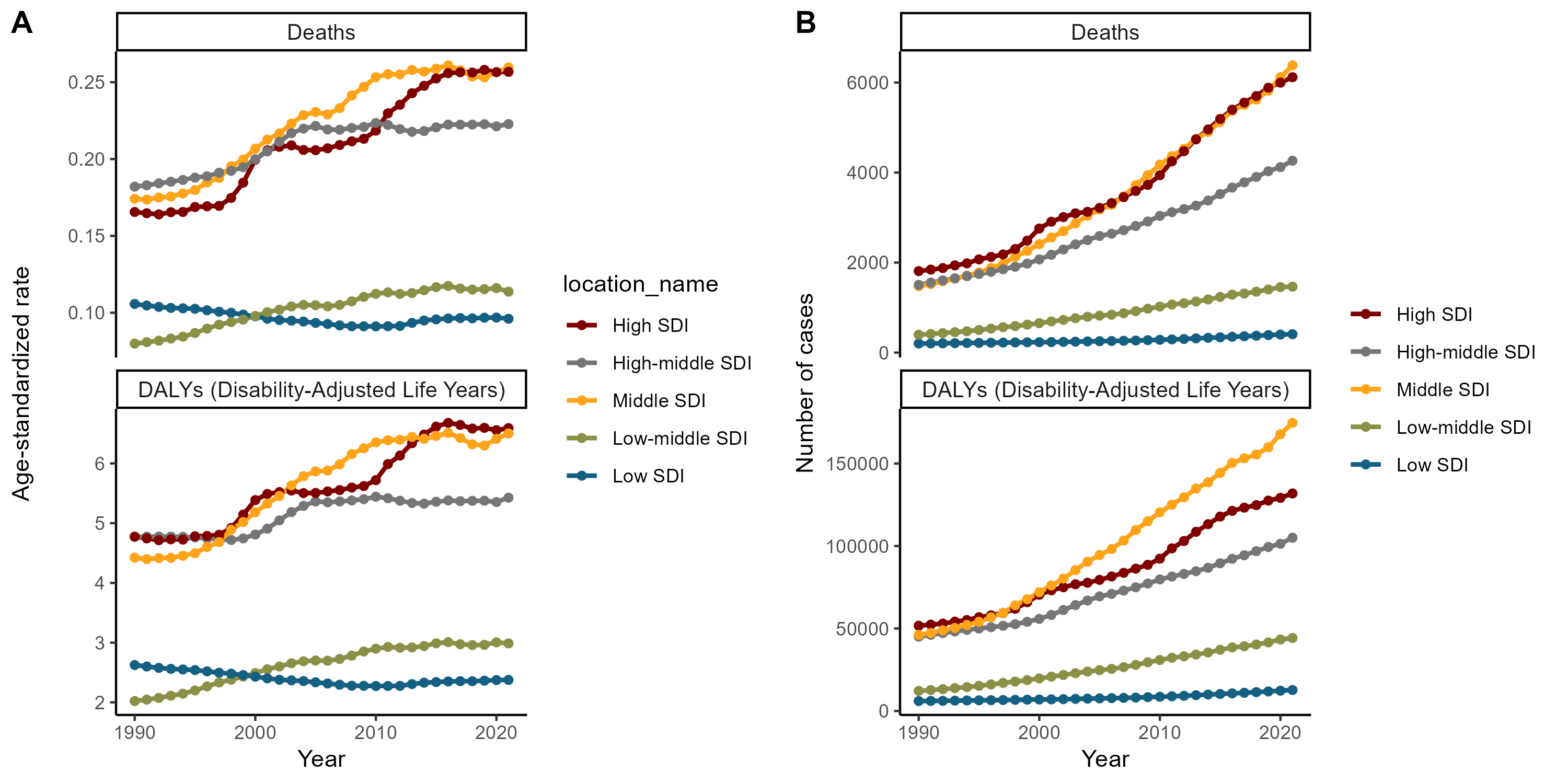

Supplement: Supplementary file 4 — Supplementary Material 4 [file 12889_2025_22560_MOESM4_ESM.png]

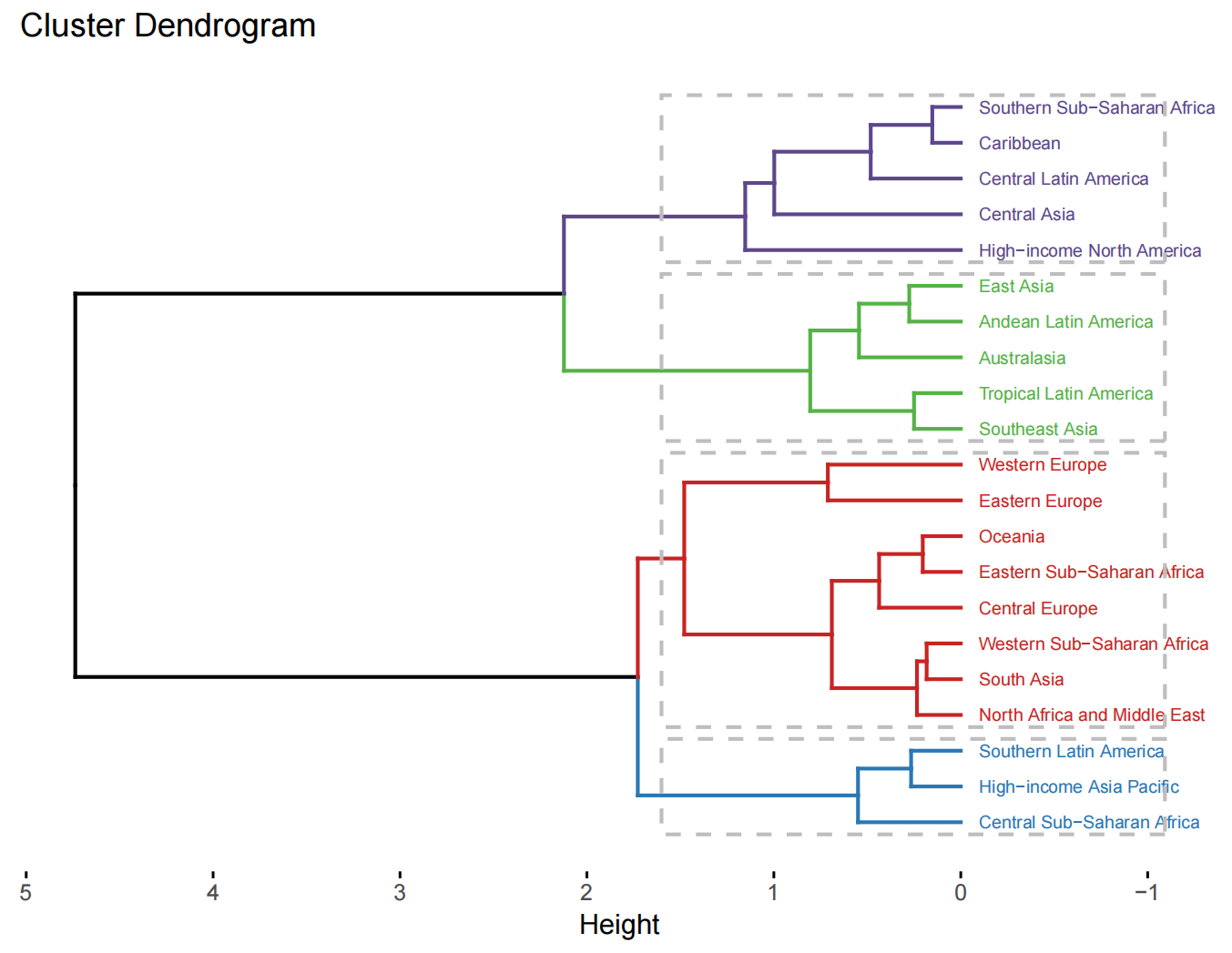

Supplement: Supplementary file 5 — Supplementary Material 5 [file 12889_2025_22560_MOESM5_ESM.png]

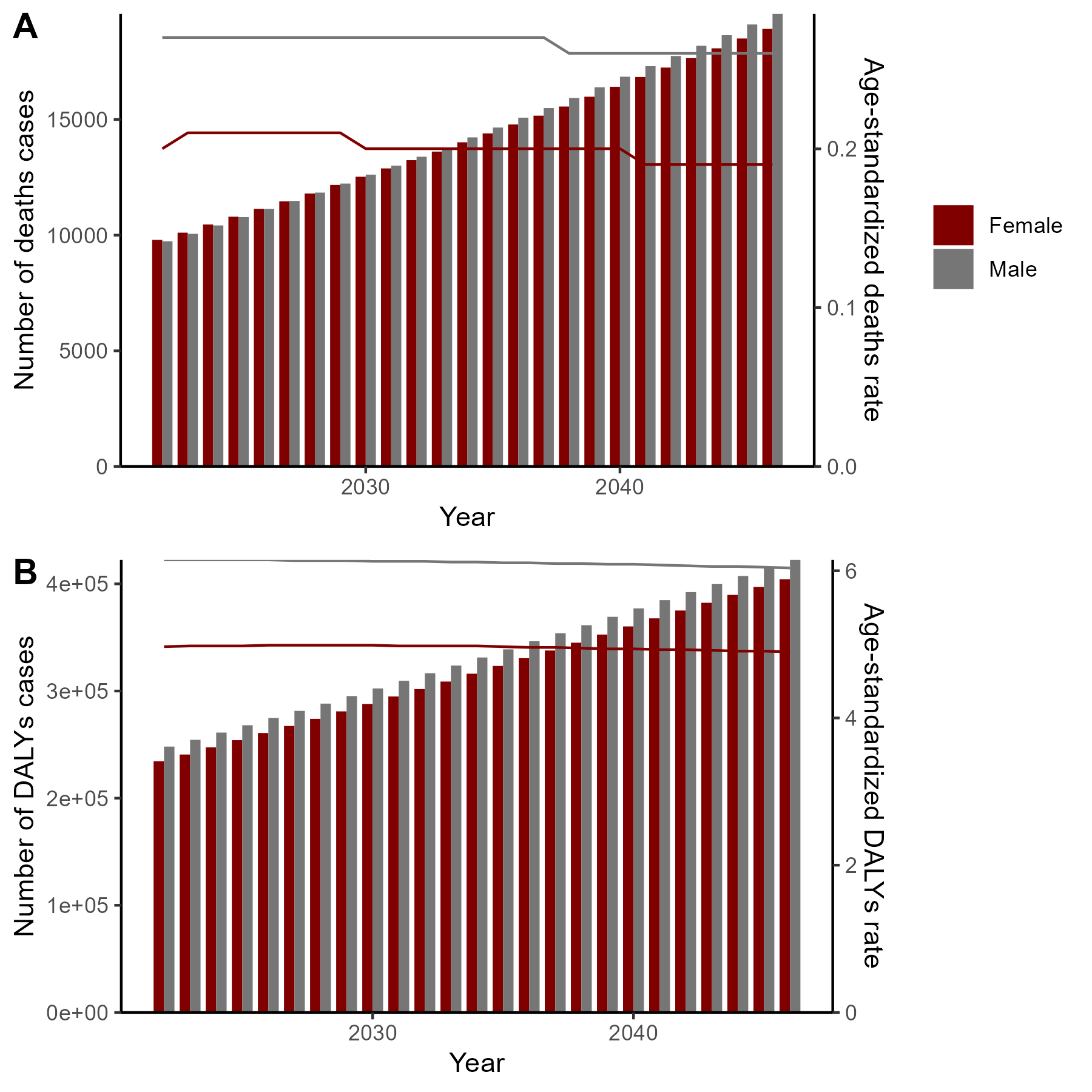

Supplement: Supplementary file 6 — Supplementary Material 6 [file 12889_2025_22560_MOESM6_ESM.png]

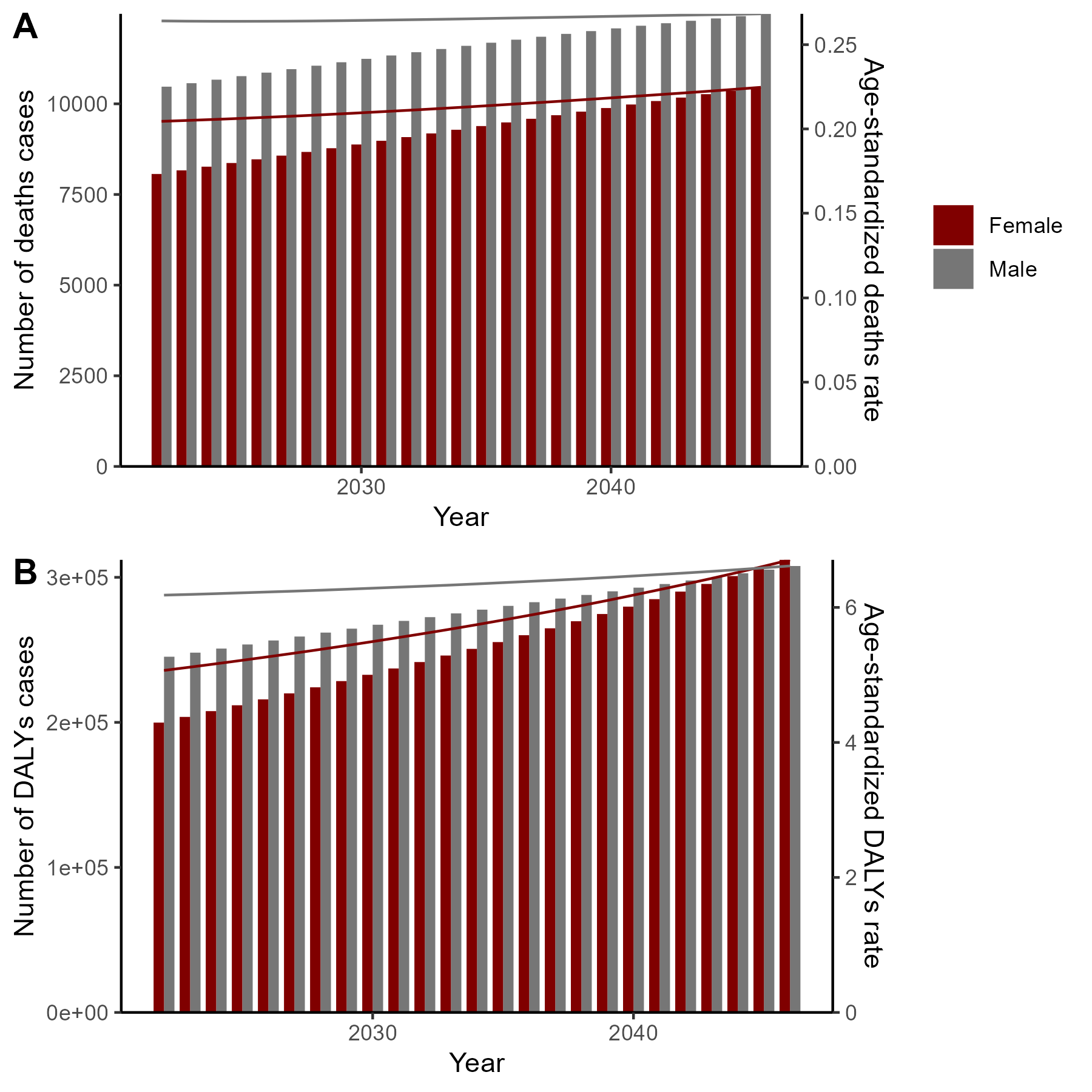

Supplement: Supplementary file 7 — Supplementary Material 7 [file 12889_2025_22560_MOESM7_ESM.png]
